# Effect of Bi, Sb, and Ti on Microstructure and Mechanical Properties of SAC105 Alloys

**DOI:** 10.3390/ma15144727

**Published:** 2022-07-06

**Authors:** Tixin Yang, Youyang Chen, Kangdong You, Ziqiang Dong, Yandong Jia, Gang Wang, Jubo Peng, Shanshan Cai, Xiaobin Luo, Chen Liu, Jiajun Wang

**Affiliations:** 1 Materials Genome Insititute, Shanghai University, Shanghai 200444, China; 372678583@139.com (T.Y.); chenyouyang@shu.edu.cn (Y.C.); yorke@shu.edu.cn (K.Y.); g.wang@shu.edu.cn (G.W.); 2 Insititute of Materials, Shanghai University, Shanghai 200444, China; jiayandong2008@163.com; 3 Yunnan Tin Group (Holding) Co., Ltd., Kunming 650000, China; jubopeng@ytc.cn (J.P.); sscai10s@alum.imr.ac.cn (S.C.); lxbinwangyi@163.com (X.L.); duke_xyz93@163.com (C.L.); wjj1209@outlook.com (J.W.)

**Keywords:** SAC solder, mechanical properties, microstructure, creep properties

## Abstract

The Sn-Ag-Cu (SAC) solder alloys with a low Ag (Ag < 3 wt.%) content have attracted great attention owing to their low cost, increased ability in bulk compliance, and plastic energy dissipation. However, some of their mechanical properties are generally lower than the SAC alloys with a higher Ag content. Adding alloying elements is an effective approach for improving the mechanical properties of the SAC alloys. In this study, the effect of Bi, Sb, and Ti on Sn-1 wt.%Ag-0.5 wt.%Cu (SAC105) solder alloys was investigated. The SAC solders with four compositions: SAC105-1 wt.%Bi, SAC105-1 wt.%Sb, SAC105-1 wt.%Bi-1 wt.%Sb, SAC105-1 wt.%Bi-1 wt.%Sb-0.4 wt.%Ti were prepared. The microstructure and phase compositions were characterized using electron scanning microscopy, and X-ray diffraction. The thermal properties and wettability were also examined. Uniaxial tensile tests and nano-indentation tests were conducted to evaluate the mechanical properties. The results show that adding Bi or Sb could increase the strength of SAC105 alloys mainly due to the solid solution strengthening effect. The creep resistance of SAC105 alloys was also improved with the additions of Bi and Sb. The co-additions of Bi and Sb into SAC105 alloys exhibit an enhanced creep resistance than that calculated by the theoretical calculation. The further addition of Ti into SAC105-1Bi-1Sb alloys demonstrated a much-improved creep resistance, which could be attributed to the synergistic effects of both solid solution strengthening and the precipitation hardening effects.

## 1. Introduction

The rapid development of the modern electronic industry imposes higher demand for the durability and reliability of solder joints [1]. However, in recent years, due to rising concern about the high toxicity of lead, researchers have attempted to develop a variety of lead-free solders to replace the traditional Sn-Pb eutectic solder [2,3]. Those lead-free solders include Sn-Ag, Sn-Cu, Sn-Ag-Cu, Sn-Zn, Sn-Bi, Sn-In, and other lead-free solders [4,5,6,7,8,9]. The lead-free solders being developed are expected to possess a comparable or better performance than that of leaded solders. The properties considered for developing the lead-free solders include environmental compatibility, material accessibility, cost, melting point, viscosity, density, thermal and electrical properties, corrosion and oxidation resistance, wettability, mechanical properties, and bonding properties. Among all the lead-free solders developed, Sn-Ag-Cu (SAC)-based solder alloys are considered to be the most promising solders, owing to their outstanding soldering properties, good mechanical properties, and excellent compatibility with the existing equipment processes, and flux chemistries [10].

Different SAC alloys have been recommended in different regions. The European Consortium Brite–Euram in the European Union has advocated the SAC alloy with a composition of Sn-3.8 wt.%Ag-0.7 wt.%Cu (SAC387), while the Japan Electronic Industry Development Association (JEIDA) recommended the alloy composition of Sn-3.0 wt.%Ag-0.5 wt.%Cu (SAC305), while the National Electronics Manufacturing Initiative in the United States suggested Sn-3.9 wt.%Ag-0.6 wt.%Cu (SAC396) [11]. However, all these SAC alloys contain higher amounts of Ag. In recent years, the SAC alloys with low silver content have attracted great attention because of the low cost, better bulk compliance, and plastic energy dissipation capability [4]. However, the low-silver SAC alloys usually show relatively lower strength and lower thermal-loading resistance than that of high-silver SAC alloys due to the reduction of Ag, which contributes to the formation of the strengthening IMC (Ag_3_Sn) phase [12]. Extensive efforts have been devoted to improving the properties of SAC-based solders.

Doping with alloying elements is an effective approach for optimizing the properties of SAC alloys [13,14,15,16]. Different alloying elements have been added to SAC solder alloys to explore their effects on influencing the microstructure and properties. Among all the alloying elements considered, Bi, Sb, and Ti are effective in improving the properties of SAC alloys. Antimony (Sb) has been proved to be an effective alloying element that can enhance the mechanical properties of solder alloys [17,18]. Adding Sb could inhibit the formation of coarse β-Sn and also refine the precipitation of Ag_3_Sn, thus improving the mechanical and thermal resistant behavior of SAC solders [18,19]. Bismuth (Bi) is another important alloying element for solder alloy that shows a significant effect in improving the creep resistance and thermal fatigue resistance [13,20,21,22]. Adding Bi can also lower the melting point and improves the wettability of SAC alloys [23]. Ti is also an important alloying element considered for optimizing the properties of lead-free solder alloys. Adding Ti into SAC solder could effectively refine the microstructure and improve the mechanical properties [24]. Although several studies have been conducted to investigate Bi, Sb, and Ti on the properties of SAC alloys, most of them only considered the effects of the sole addition of one of the alloying elements. Few studies examined the alloying effect of simultaneous additions of Bi, Sb, and Ti on the microstructures and properties of low-silver SAC alloys. In this study, a systematic investigation of the addition of Bi, Sb, and Ti on the melting properties, wettability, microstructure, and mechanical properties of the low-silver solder Sn-1.0Ag-0.5Cu (SAC105) has been conducted. The results demonstrate that an enhanced creep resistance could be derived by the additions of Bi, Sb, and Ti into the SAC105 alloy. The affecting mechanism of the alloying element on the properties of SAC105 was discussed.

## 2. Experimental Methods

### 2.1. Processing of Lead-Free Solder

A series of SAC solder alloys with different compositions, as listed in Table 1, were synthesized using a vacuum induction melting furnace (KYKY WK-ⅡAcuum Induction Melting Furnace). Pure tin (99.99 wt.%), silver (99.99 wt.%), copper (99.99 wt.%), Bismuth (99.9 wt.%), Antimony (99.9 wt.%), and Titanium (99.9%) were used as raw materials. The melting process is carried out in a vacuum induction furnace under a high purity argon atmosphere. The melting process is held at 800 °C for 1 h, then poured into a steel mold to prepare the ingots with a size of 50 mm × 20 mm × 10 mm.

### 2.2. Characterization of Physical Properties

#### 2.2.1. Melting Properties

A synchronous thermal analyzer (STA, STA 2500 Regulus, NETZSCH, Selb, Germany) was used to test the melting properties of the solder alloys. The tests were conducted using a sample with a weight of 15–25 mg which was placed in an alumina crucible under an argon atmosphere. The sample was heated up to 260 °C with a heating rate of 5 °C/min and then was cooled to 30 °C with a cooling rate of 5 °C/min.

#### 2.2.2. Wettability Test

The wettability test was performed using a copper plate with a size of 3 mm × 3 mm × 3 mm as the substrate. The surface of the copper plate was ground using emery papers up to 3000 grade; then, it was cleaned using alcohol in an ultrasonic water bath. The mass of the solder ball used for the wettability tests was 0.3 g. The solder flux of RMA218 from Seamark ZM was used for the wettability test. A high-temperature, wetting angle tester (OCA25HTV, DataPhysics Instruments, Stuttgart, Germany) was used for wetting experiments. The experimental temperature was increased from room temperature with a heating rate of 5 °C/min to 500 °C, then was held for one hour, and was decreased to room temperature with a cooling rate of 5 °C/min. The wettability tests were performed three times for each alloy composition.

#### 2.2.3. Microstructural Characterization

The microstructure examination was performed using Scanning Electron Microscopy (HITACHI FlexSEM 1000 II, HITACHI, Tokyo, Japan) equipped with an Energy Dispersive X-ray Spectrometry (Bruker Esprit Compact). The phase analysis was performed utilizing an X-ray Diffractometer (Bruker D2 Phaser) using Cu-Kα radiation with an accelerating voltage of 40 kV. The scanning step was set to 0.02° across the angle (2θ) range of 20–85°.

### 2.3. Mechanical Properties

**Figure 1:**
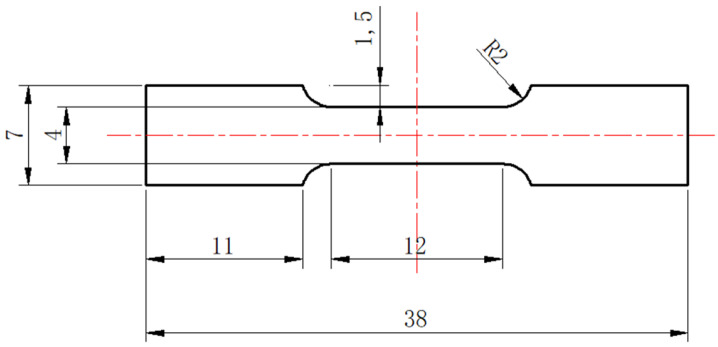
Illustration of the tensile test sample.

The tensile tests were carried out using a mechanical testing tester (CMT-5205, SANS, Shenzhen, China) with a constant strain rate of 3 × 10^−3^ s^−1^. As shown in Figure 1, the tensile specimens in a dog-bone flat shape with a thickness of 2 mm and a gauge length of 20 mm were from the as-cast ingots. For each composition, at least five parallel samples were tested. SEM was used to observe the fracture morphology of tensile specimens after tensile tests.

### 2.4. Creep Properties

All solder samples were ground and polished to produce a smooth surface for creep tests. The nano-indentation tests were performed at room temperature using a micro-indentation machine (KLA i-micro, KLA, San Francisco, California, USA). The samples were fixed on the loading table and placed in the nano-indentation apparatus. The maximum loading force for the creep test was 30 mN. The holding time at the maximum loading force was 300 s. More than five repeated tests were performed at different regions for each sample.

## 3. Results

### 3.1. Thermal Analysis

**Figure 2:**
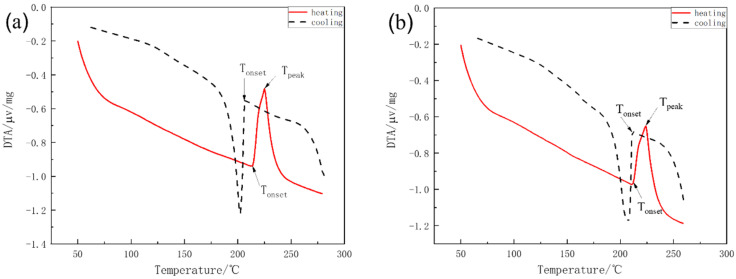

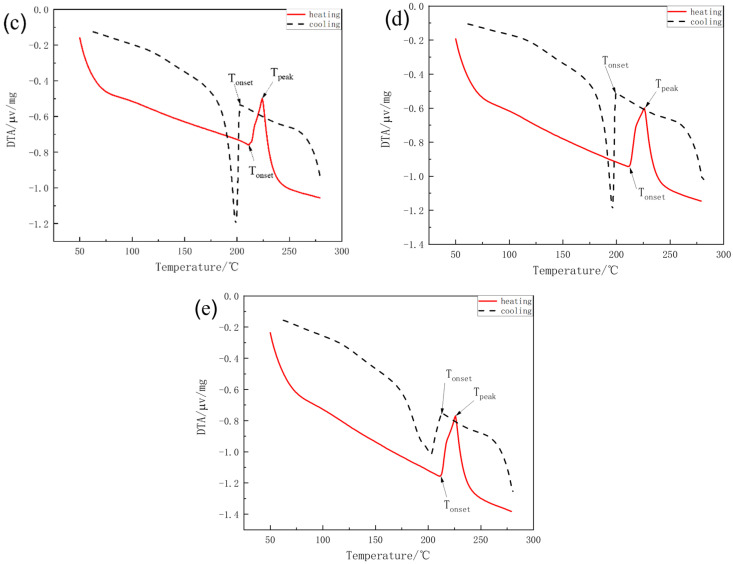
DTA curves of (**a**) SAC105, (**b**) SAC105-1Sb, (**c**) SAC105-1Bi, (**d**) SAC105-1Bi-1Sb, (**e**) SAC105-1Bi-1Sb-0.4Ti.

Melting temperature is one of the most vital properties of solder alloys. A low melting temperature and a narrow pasty range are desirable for enhancing the reliability of solders [4]. A higher melting temperature increases the soldering temperature and, thus, adversely affects the quality of electronic boards and polymer devices. Moreover, the increased soldering temperature might lead to the formation of thicker intermetallic compounds at the interface and hence reduces the bonding strength [10,25]. Differential thermal analysis (DTA) was conducted to measure the melting properties of the prepared alloys. The degree of undercooling obtained in DTA is defined as the difference between the temperature at the onset of the heating reaction peak and the temperature at the onset of the cooling reaction peak [23]. The temperature difference shows the degree of undercooling required for nucleation during the solidification process. The pasty range is defined as the difference between the onset and peak temperatures of the heating reaction peak [24]. Figure 2 shows the DTA curves measured for the five solder alloys, and the results were summarized in Table 2. As shown in Table 2, the T_onset_ of the cooling is almost similar for all the alloys prepared, which fall into the range of 211 °C~214 °C. Introducing the alloying elements slightly reduced the T_onset_ of the cooling by 2–3 °C, as shown in Table 2. The alloying elements affect the T_onset_ of the heating more significantly. The addition of 1 wt.% of Bi into SAC105 reduced the temperature from 206.6 °C to 202.8 °C, and its undercooling range increased slightly from 7.4 °C to 8.8 °C. Conversely, adding 1 wt.% Sb increased the T_onset_ of the heating to 211 °C accompanied by a decrease in the undercooling to 1.2 °C. When Bi and Sb were simultaneously added, the T_onset_ of the heating was decreased to 200 °C, indicating the dominant effect of Bi in affecting the solidus temperature. Meanwhile, the undercooling was increased to 11.7 °C. When Bi, Sb, and Ti were added to the SAC105 alloy, the undercooling degree decreased to 0.4 °C, due to the obvious increase in T_onset_ of the heating by the addition of Ti. The narrow melting range presented by the SAC105-1Bi-1Sb-0.4Ti alloy should be helpful to form reliable joints since the duration of time that alloy is present as a partial liquid is greatly reduced [26]. The pasty ranges of SAC105-1Bi and SAC105-1Bi-1Sb were elevated compared to SAC105, while both SAC105-1Sb and SAC105-1Bi-1Sb-0.4Ti showed a decrease in their pasty ranges compared to SAC105.

### 3.2. Wettability Test

**Figure 3:**
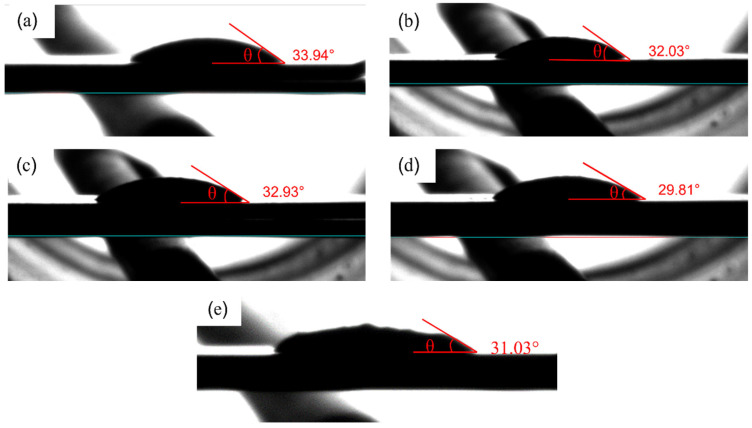
Wetting angle of (**a**) SAC105, (**b**) SAC105-1Sb, (**c**) SAC105-1Bi, (**d**) SAC105-1Bi-1Sb, (**e**) SAC105-1Bi-1Sb-0.4Ti on Cu substrate.

**Figure 4:**
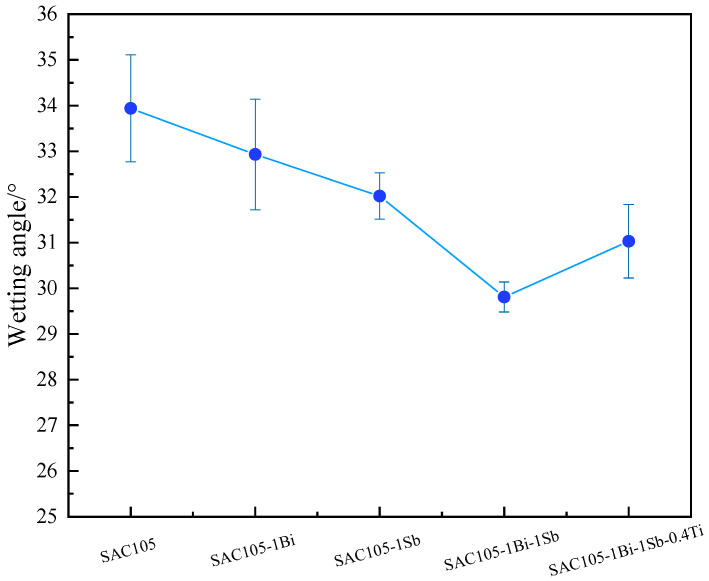
Contact angle of SAC105, SAC105-1Bi, SAC105-1Sb, SAC105-1Bi-1Sb, SAC105-1Bi-1Sb-0.4Ti on Cu substrate.

The wettability of lead-free solder alloy was determined by measuring the contact angle (°) of solder alloys on Cu substrates. The wettability test was performed three times for each alloy composition. A smaller contact angle indicates a better wettability of the solder alloy. The wettability of materials can be divided into the following four categories: (1) excellent wetting if the contact angle is between 0°–30°; (2) good wetting if the contact angle is between 31°–40°; (3) admissible wetting if the contact angle is between 41°–55°; (4) unacceptable wetting if the contact angle is between 56°–70° [26]. To get good wettability, the contact angle θ should be between 0° and 45°. The measured contact angles of solder alloys are shown in Figure 3 and Figure 4. It can be seen that adding either Bi or Sb reduced the contact angle and, hence, improved the wettability. The beneficial effect became more significant when Bi and Sb were added simultaneously. However, further introducing Ti into SAC-1Bi-1Sb reduced the wettability. Wetting is a process with the competition between internal forces (interatomic forces of the liquid) and external forces (between the liquid and the substrate). As the internal forces increase (stronger bonding), the liquid prefers to maintain the interatomic bonding without bonding to the substrate surface, resulting in a large wetting angle [23]. Conversely, if the molten inner bonds become weaker, wettability could be improved. When Bi or Sb are added into SAC solder, weaker Sn-Bi or Sn-Sb bonds could be formed instead of Sn-Sn bonds. Therefore, the inner bonds were weakened; hence, the wettability could be improved, whereas, Ti, which is an active element, could cause an increase in the internal forces. Therefore, when Ti was introduced into the SAC105-Bi-Sb alloy, the wettability was slightly reduced.

### 3.3. Microstructure Examination

**Figure 5:**
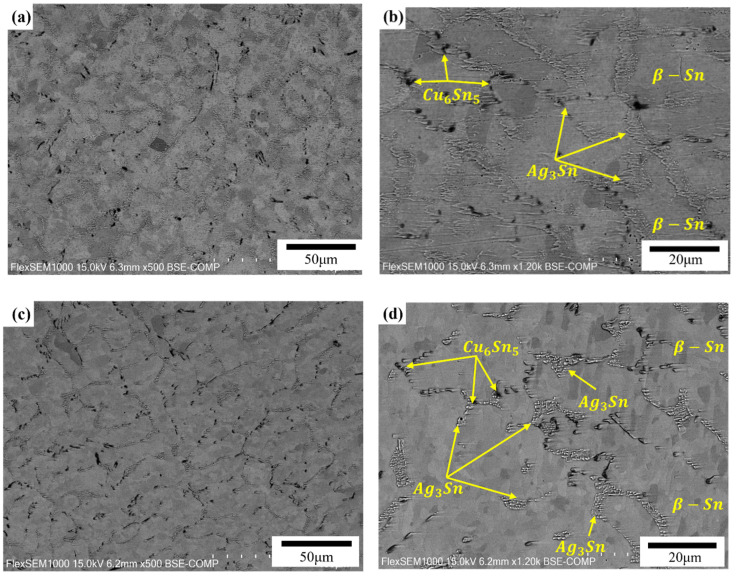

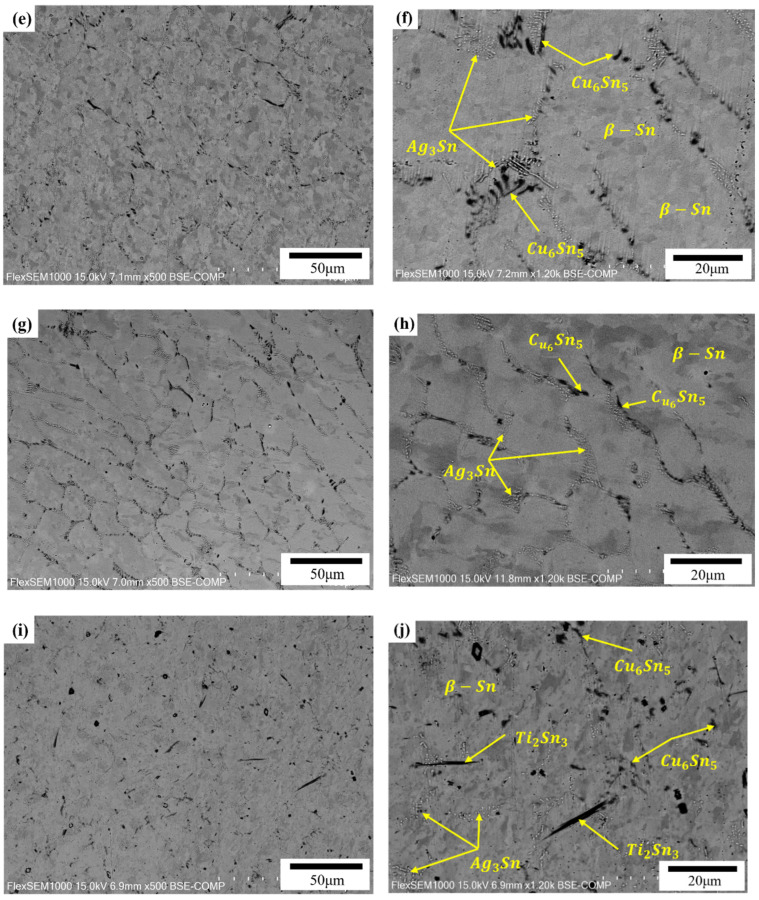
Microstructure of (**a**,**b**) SAC105, (**c**,**d**) SAC105-1Bi, (**e**,**f**) SAC105-1Sb, (**g**,**h**) SAC105-1Bi-1Sb, (**i**,**j**) SAC105-1Bi-1Sb-0.4Ti. (**a**,**c**,**e**,**g**,**i**): 500×, (**b**,**d**,**f**,**h**,**j**): 1200×.

**Figure 6:**
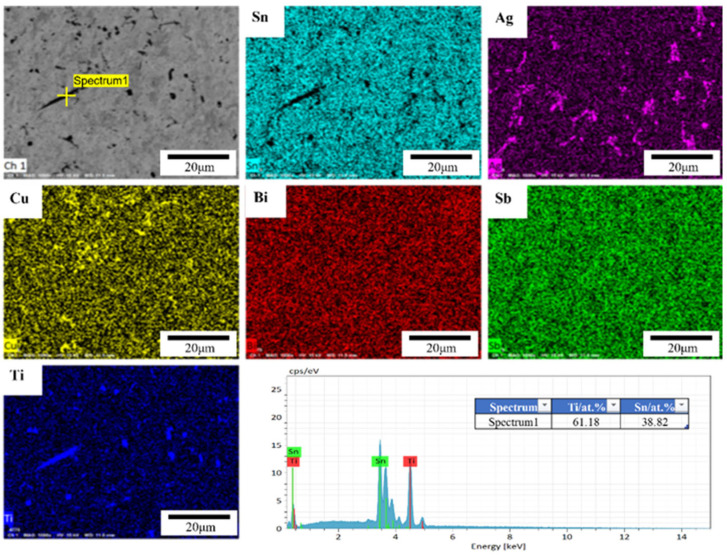
EDS elemental mapping of SAC105-1Bi-1Sb-0.4Ti and EDS quantitative analysis result of Ti_2_Sn_3_ IMC.

Figure 5 shows the SEM images of all the alloys. As shown in Figure 5, the microstructure of the SAC alloys consists of the pro-eutectic β-Sn dendrites with the eutectic phase dispersed. The eutectic phase is composed of Ag_3_Sn and Cu_6_Sn_5_ intermetallic compounds distributed in the Sn matrix. When 1 wt.% Bi or Sb were added to SAC105, Bi or Sb were predominately dissolved into the Sn matrix, and a slightly evident change in microstructure was observed, as shown in Figure 5. Compared with the SAC105 alloy, the difference for Bi and Sb added alloys is that more uniform net-shape distributed IMCs appeared in the alloy. Meanwhile, the size of β-Sn grains was changed, due to the additions of Bi and Sb. The average size of β-Sn grains was determined by measuring more than 20 grains in each alloy, and the results are listed in Table 3. Please note that the grain size of SAC105-1Bi-1Sb-0.4Ti is hard to be determined by the microstructure examination and, hence, is not listed. It can be seen that the refinement effect of Bi is more pronounced. Meanwhile, the size difference between Bi and Sn is larger than that of Sb and Sn. Therefore, the solution strengthening effect caused by the Bi addition should also be stronger than that of the Sb addition. Therefore, it can be expected that the strength increase brought about by the addition of Bi will be more significant than that caused by the addition of Sb. When Ti was introduced into the SAC105-1Bi-1Sb alloy, needle-shaped precipitates were presented, as shown in Figure 5. Meanwhile, the IMCs (Ag_3_Sn and Cu_6_Sn_5_) were distributed more homogeneously in the Sn matrix. Figure 6 shows the EDS analysis of the SAC105-1Bi-1Sb-0.4Ti alloy. As shown in Figure 6, the distributions of Bi and Sb are quite uniform. The results show that the needle-shaped precipitates mainly consist of Ti and Sn. According to the phase diagram of Sn-Ti [27] and Reference [28], the precipitates were identified as Ti2Sn3, which was formed due to the reaction between Sn and Ti.

**Figure 7:**
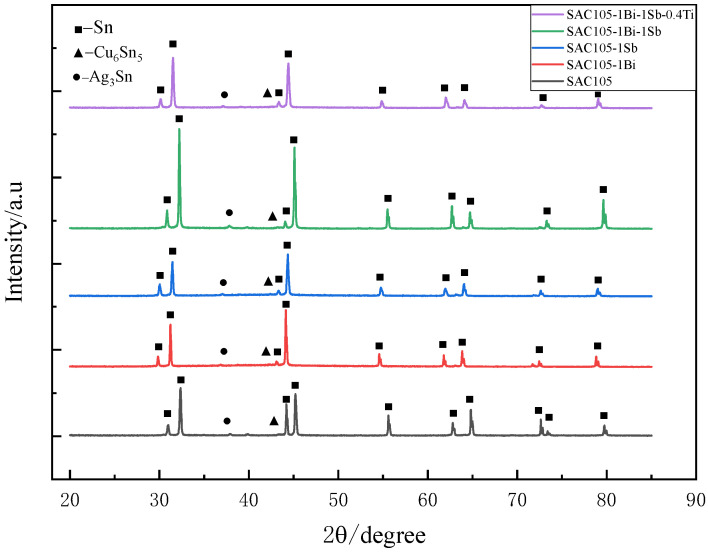
XRD patterns of SAC105, SAC105-1Bi, SAC105-1Sb, SAC105-1Bi-1Sb, SAC105-1Bi-1Sb-0.4Ti.

Figure 7 shows the XRD patterns obtained for all the prepared alloys. All the alloys consist of three major phases: β-Sn phase, Cu_6_Sn_5,_ and Ag_3_Sn phases. It is worth noting that the XRD peaks shifted left when 1 wt.% Bi or Sb were introduced into SAC105 alloy. The shifts might be attributed to the lattice distortion caused by the incorporation of the Bi or Sb atoms, which are larger than the Sn atom. However, the existence of Bi-, Sb-, and Ti-bearing compounds could not be identified by the XRD analysis, due to their low contents [24].

### 3.4. Mechanical Property

**Figure 8:**
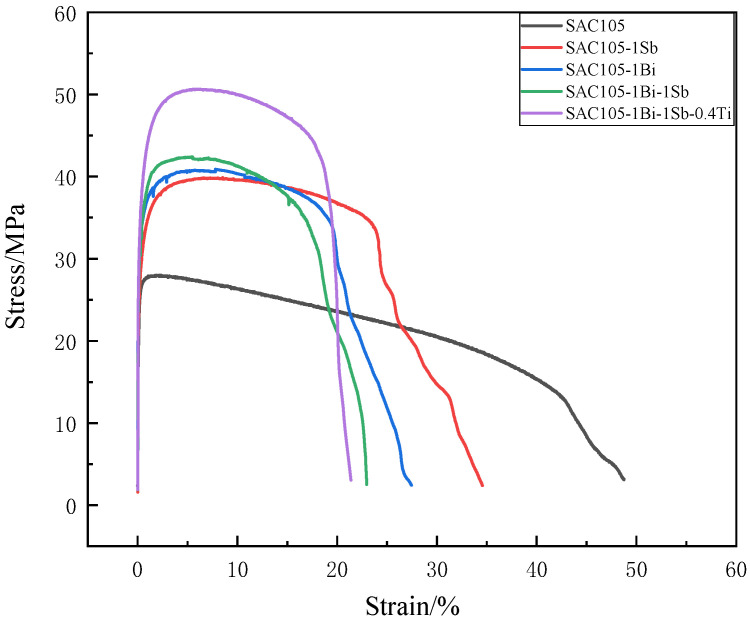
Comparison of mechanical properties of solder alloys with different compositions.

The tensile properties measured for the prepared alloys are shown in Table 4 and Figure 8. The results show that adding 1 wt.% of either Bi or Sb could improve the tensile strength by almost 50% of that measured for SAC105. The improvements in the tensile strength are almost similar for the two samples SAC105-1Bi and SAC105-1Sb. However, alloy SAC105-1Sb shows higher ductility than that of alloy SAC105-1Bi, indicating that adding Sb is beneficial to preserving the ductility compared to the addition of Bi. The strengthening effect of the co-additions of Bi and Sb is almost similar to the single addition of 1 wt.% Bi or Sb. No overlaid effects were observed for the co-additions of Bi and Sb. With the further addition of 0.4 wt.% of Ti into the SAC105-1Bi-1Sb alloy, the tensile strength was further enhanced to 50.66 MPa, which is almost twice the strength of the SAC105 alloy. Meanwhile, the elongation was reduced to 21.35%.

Both Bi and Sb are the solid solution strengthening elements for Sn solder alloys. Per the Sn-Bi binary phase diagram, the solubility of Bi in β-Sn is at about 3 wt.% at ambient temperature [29]. Bi with the amount of 1 wt.% could be completely dissolved into the β-Sn matrix, thereby enhancing the strength of solder alloy due to the solution strengthening effect. Adding Bi also refines the microstructure as shown in Table 3. Meanwhile, the addition of Bi also embrittles the bulk solder, as reflected by the significant reduction of elongation from 48.75% (SAC105) to 27.42% (SAC105-1Bi). Adding 1 wt.% of Bi decreases the melting point (Table 2) and increases the wettability (Figure 3), which is beneficial to the improvement of the solderability. Adding 1 wt.% of Sb has a similar effect to that of the Bi addition. Sb could be dissolved into the β-Sn matrix, which strengthens the SAC105 solder. Although it does not refine the matrix as well as Bi, it can retain higher plasticity compared to Bi. According to the Sn-Sb binary phase diagram, the Sb-containing precipitates could only be formed in SAC alloy when the Sb content is greater than 3 wt.% [30]. No Sb-containing precipitates were observed in the alloys of this study.

It is worth noting that the reduction of the elongation caused by the addition of Sb is less than the Bi added alloy (SAC105-1Bi). This should be attributed to the microstructure variation caused by the alloying effect of different elements. In previous studies, the addition of Bi and Sb can generally suppress the coarsening of the β-Sn and IMCs and refine them [19,23]. Therefore, it is reasonable that the Bi, and Sb both significantly improve the strength of the solder alloy. When Bi, Sb, and Ti were simultaneously added, the original intermetallic compounds in the microstructure were refined, and the tensile strength was enhanced. The EDS pattern shows that Bi, Sb does not precipitate and form intermetallic compounds, due to its small content. Ti could form a Ti_2_Sn_3_ intermetallic compound that is distributed in a long-strip shape in the Sn matrix. The presence of the IMC precipitates could be beneficial to the improvement of the tensile strength of SAC105 alloy, owing to the precipitation-strengthening effect. However, the existence of large IMC particles could also reduce ductility.

**Figure 9:**
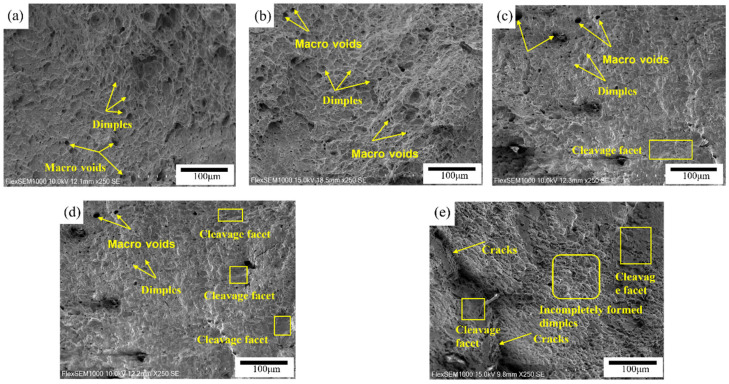
SEM of fracture surfaces of solder alloys: (**a**) SAC105, (**b**) SAC105-1Sb, (**c**) SAC105-1Bi, (**d**) SAC105-1Sb-1Bi, (**e**) SAC105-1 Sb-1Bi-0.4Ti.

The fracture topography of the alloy samples after uniaxial tensile tests were examined using SEM is shown in Figure 9. Ductile dimples and voids are the major features that appeared on the surface of samples SAC105 and SAC105-1Sb, which is consistent with the good ductility exhibited. With the addition of 1 wt.% Bi, portions of cleavage facets with large holes were presented, indicating the reduced ductility. Figure 9d shows the fracture morphology of SAC105-1Bi-1Sb, which consists of dimples, cleavage facets, and several larger holes, indicating combined failure features of plastic fracture and brittle fracture. The fracture morphology of sample SAC105-1Bi-1Sb-0.4Ti contains amounts of cleavage areas with a few dimples and cracks appearing. Overall, the fracture topography of the samples is generally consistent with the tensile testing results.

### 3.5. Creep Performance

**Figure 10:**
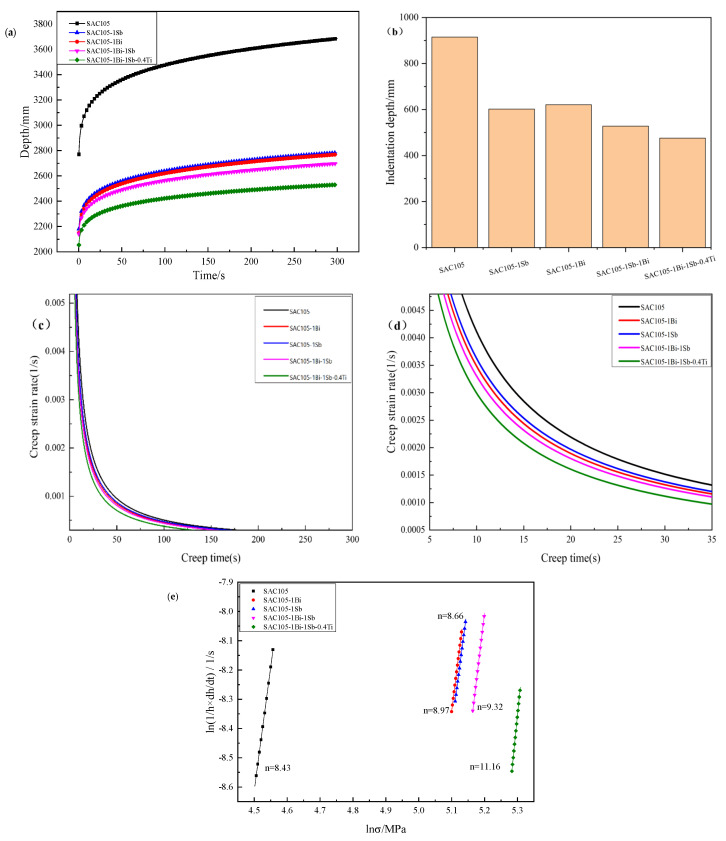
(**a**,**b**) The penetration depth in different solder alloys, (**c**,**d**) Creep strain rate of solder alloys with different compositions for (**c**) 0–300 s, (**d**) 5–35 s, (**e**) creep, stress exponent of solder alloys with different compositions.

The nano-indentation technique has been utilized in assessing the creep properties of solder alloys in many reported studies. The penetration of a sharp indenter could form an instantaneous strain field underneath the indenter. The indentation creep rate could be derived based on the moving rate of the elastic/plastic boundary of the strain field toward the materials. Creep properties were characterized using nano-indentation tests and the results are shown in Figure 10. The change in indentation depth is considered an indicator of creep performance [31]. As shown in Figure 10b, the indentation depth during the holding time decreased with the subsequent addition of the alloying elements, indicating the improvement of the creep resistance. SAC105 alloy shows the deepest initial depth and the deepest indentation depth. The starting depth and the indentation depths of the alloys SAC105-1Bi and SAC105-1Sb were quite similar, indicating that the mechanical responses of those two alloys were quite similar. SAC105-1Bi-1Sb alloy showed higher creep resistance than the alloys adding Bi or Sb alone. Nevertheless, it was noted that the alloy SAC105-1Bi-1Sb-1Ti exhibited the highest creep resistance among all the alloys tested.

Creep strain rate is another important factor for creep performance [31], which is defined as (1/h) (dh/dt) based on the curve of penetration depth. It can be seen from Figure 10c,d that the creep strain rate gradually decreases with the testing time. The solder alloys with the addition of alloying elements show lower strain rates than that of SAC105, which is consistent with the depth variation in indentation. The creep strain rates decrease in the order of SAC105, SAC105-1Sb, SAC105-1Bi, SAC105-1Bi-1Sb, and SAC105-1Bi-1Sb-0.4Ti.

Figure 10e shows the creep stress exponent for the solder alloys with different compositions, which was derived by comparing the logarithm of the creep strain rate (1/h) (dh/dt) with the logarithm of the stress σ. The creep stress exponent n values were derived by linear regression fitting [32]. As can be seen from the figure, the n value of SAC105 is 8.34. Adding alloying elements to SAC105 alloys increases the creep stress index. Among them, SAC105-1Bi-1Sb-0.4Ti has the largest n value of 11.16. The next one is SAC105-1Bi-1Sb with an n value of 9.32, and SAC105-1Bi and SAC105-1Sb with n values of 8.97 and 8.66, respectively. Therefore, incorporating more, different alloying elements is beneficial to the improvement of creep resistance. Different creep mechanisms have been proposed in the literature for the interpretation of the creep phenomenon of lead-free solders, which include bulk and grain boundary diffusion, dislocation climbing and gliding, grain boundary sliding, etc. [31,32]. It was reported that when n is less than 3, grain boundary sliding dominates the creep deformation. When n is greater than 3, the dislocation climbing and gliding is the major acting mechanism [33]. Therefore, the incorporation of any factors that can affect the dislocation movement would influence the creep. Incorporation of Bi, Sb, and Ti can activate two determinative strengthening mechanisms: (1) The precipitates, i.e., Ag_3_Sn, Cu_6_Sn_5_ and Ti_2_Sn_3_, can restrict the glide/climb and cross-slip of dislocations; (2) The solutes exited in the matrix can drag the dislocations. Therefore, the samples SAC105-1Bi and SAC105-1Sb show very similar creep stress exponents due to the fact that the concentrations of the alloying elements are very similar, and the volumes of IMCs precipitates are almost identical. Alloy SAC105-1Bi-1Sb exhibits a larger creep stress exponent than that of SAC105-1Bi or SAC105-1Sb due to the increased solutes which can drag the dislocation motions. The further addition of Ti in the Sn solder introduces additional needle-like Ti_2_Sn_3_ precipitates which could restrict the glide/climb and cross-slip of dislocations. Therefore, the creep resistance was further improved.

## 4. Discussion

Pure tin is a soft metallic material with low values for tensile strength. The tensile strength of pure tin is only 17 MPa~21 MPa. Therefore, various alloying elements are usually introduced into the tin metal to optimize its metallic properties. The two dominant strengthening mechanisms for Sn solder metals, by adding alloying elements, are solid solution strengthening and precipitation strengthening.

Bi and Sb are the solid solution additive elements in Sn-Ag-Cu system alloys and, thus, improve the tensile strength by the solid solution effect. The dissolving of Bi or Sb into the Sn matrix could induce the lattice distortion in the crystalline structure which could act as an obstacle to impede the movement of dislocations. The impeding force could be represented using the following equations [34]:

(1) $$F_{m}=\mu br_{s}\left|\varepsilon \right|$$

(2) $$\varepsilon =\frac{r_{s}-r_{m}}{r_{m}}$$

where ε is the misfit strain, $r_{s}$ is the solute atomic radius, $r_{m}$ is the atomic radius in the matrix phase, $F_{m}$ is the maximum force of the obstacle acting on a dislocation, μ is the modulus of rigidity, and b is the Burgers vector.

Hence, the impeding force is proportional to the misfit strain associated with the atomic radius of the solute (Bi or Sb) and the matrix (Sn). The impeding force should also be affected by the number of solute atoms that participate in blocking the motion of dislocation. Obviously, the solid strengthening effect is associated with the size difference between the solute and matrix atoms, as well as the concentration of the solute element. The sizes of the different elements considered in this study are listed in Table 5.

The solution strengthening effect is the dominant effect for Bi and Sb, due to its large solubility in the Sn matrix. Since the size difference between Bi and Sn is larger than that of Sb and Sn, the strengthening effect of adding Bi is more evident than that of Sb. This could be demonstrated by the higher strength of SAC105-1Bi than that of SAC105-1Sb. The addition of Bi and Sb also increases the creep strength, due to the solid solution strengthening. If it is assumed that multiple solute atoms are involved in the basic process of one-time differential row motion, the increase in the creep strengthening rate that comes from the solid solution strengthening could be calculated using the Labusch limit [35]:

(3) $$S=|\varepsilon {|}^{\frac{4}{3}}\cdot C^{\frac{2}{3}}+1$$

where *C* is the concentration of solute atoms.

**Figure 11:**
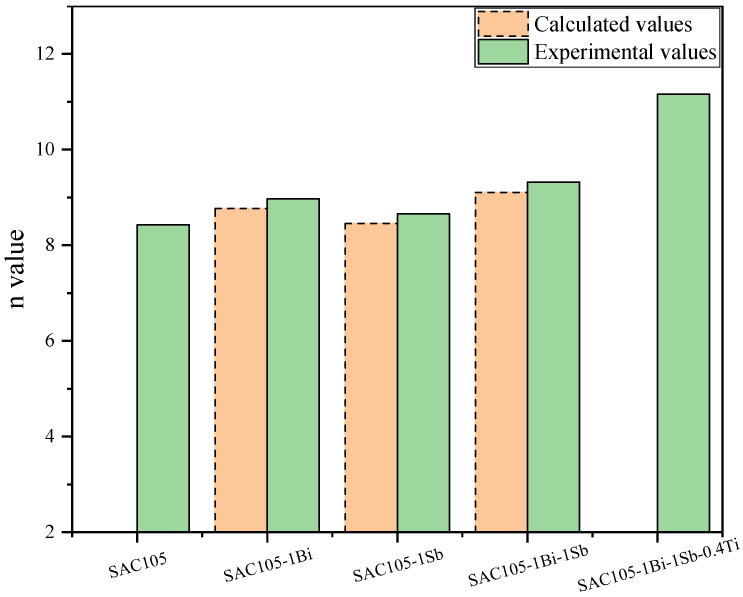
Comparison of the calculated and experimental values.

The S values were calculated to be 1.04 and 1.0034 for Sn added with Bi and Sb of 1 wt.%, respectively [35]. Therefore, the creep stress exponent n could be calculated to be 8.43 for SAC105, 8.78 for SAC105-1Bi, and 8.46 SAC105-1Sb, respectively. A comparison of the calculated and experimental values is shown in Figure 11. For SAC105 and SAC105-1Bi-1Sb-0.4Ti, only experimental values are put into the figure for comparison. As can be seen in the graph, the calculated stress exponents are smaller than the experimental values. The possible reason might be that only the solid solution strengthening effect is considered in the calculation. The additions of Bi and Sb also refine the microstructure as shown in the microstructure examination. For the SAC105-1Bi-1Sb alloy sample, it is not clear whether it is a linear superposition of their respective roles or if one element plays a dominant role. Therefore, the S values were calculated to be from 1.0066 to 1.0815 by assuming the addition of Bi or Sb with 2 wt.% [35]. This corresponds to the n values of 8.486 (SAC105-2Sb) and 9.104 (SAC105-2Bi). However, the experimental n value determined for SAC105-1Bi-1Sb is 9.32 which is even larger than the calculated n of SAC105-2Bi, which means the enhancement in the creep resistance caused by the co-additions of Bi and Sb is more evident than that of the additions of Bi at the same concentration level. Since the solid solution effect of Bi is obviously larger than that of Sb, the enhancement of the creep resistance should not solely originate from the solid solution effect. Other effects, i.e., grain refinement could also contribute to the improvement of the creep resistance for the SAC105 co-added with Bi and Sb. When Bi, Sb, and Ti were added simultaneously, the experimental n value of SAC105-1Bi-1Sb-0.4Ti reached 11.16, which is much larger than that of SAC105-1Bi-1Sb at 9.32. The further enhancement of creep resistance should be attributed to the precipitation-strengthening effect caused by the formation of Ti_2_Sn_3_ intermetallic. Obviously, the precipitation-strengthening effect is greater than the solid solution strengthening effect.

**Figure 12:**
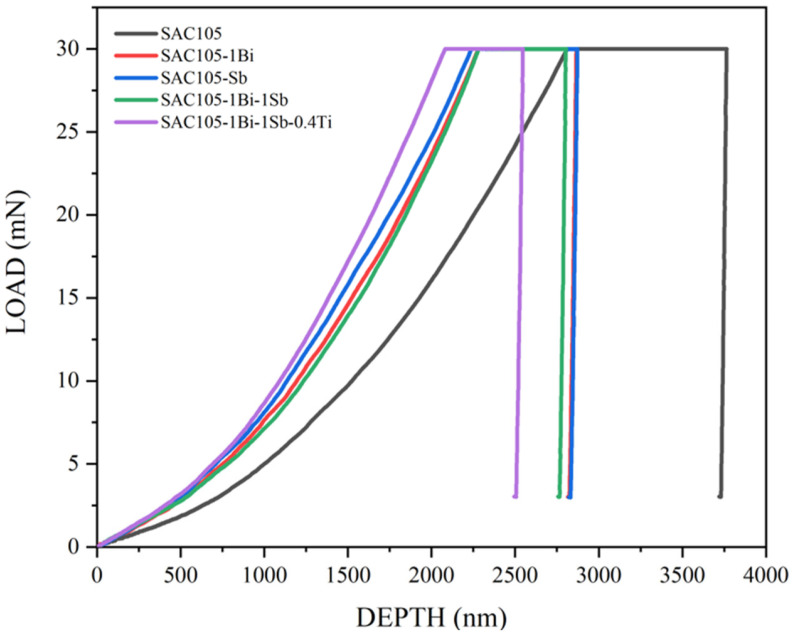
The indentation load-depth curves of all alloys.

All the results demonstrate that much-enhanced mechanical properties could be achieved by the simultaneous additions of Bi, Sb, and Ti. This could be further confirmed by the nano-indentation test. Figure 12 shows the indentation load-depth curves of the alloy samples tested. Take the curve ABCD for sample SAC105-1Bi-1Sb-0.4Ti as an example. The AB portion represents the loading process, and the CD portion is the unloading process. The portion of BC depicts the depth change in indentation under the peak load, i.e., a creep process occurring during the holding time. Obviously, the SAC105 sample shows a loading-unloading curve at the position with a larger depth of indentation. The curves of SAC105-1Bi, SAC105-1Sb, and SAC105-1Bi-1Sb are quite close to each other. The curve of SAC-1Bi-1Sb-0.4Ti shifts to the left with a smaller indentation depth. All the results demonstrate that an evident optimized mechanical property could be achieved by the simultaneous additions of Bi, Si, and Ti.

The effects of various alloying elements, such as Bi, Sb, etc., on the properties of SAC105 alloys have been investigated by several researchers. Chen et al. [35] found that with the addition of 1 wt.% Bi, the tensile strength of SAC105 alloy increased from 30.80 MPa to 45.28 MPa, while the elongation decreased from 35.92% to 20.09%. Hammad et al. [12] found that when 0.5 wt.% Sb was introduced into SAC105 alloy, the tensile strength could be improved from 19.7 MPa to 30.1 MPa, and the calculated creep stress index was increased from 8.6 to 10.7. The current study demonstrates that when Bi, Sb, and Ti are simultaneously added into SAC105, the tensile strength could be evidently increased from 27.98 MPa to 50.66 MPa. Meanwhile, the creep properties are also greatly improved, and the creep stress index increased from 8.43 to 11.16. The significant improvement of the mechanical properties could be attributed to the synergistic alloying effects of Bi, Sb, and Ti, which contribute to the improvement of mechanical properties via both solid solution strengthening and precipitation-strengthening effects.

## 5. Conclusions

In this study, we investigated the alloying effect of three elements, Bi, Sb, and Ti, on the microstructure and properties of SAC105 solder alloys. The microstructure, physical properties, and mechanical properties of SAC105, SAC105-1wt.%Bi, SAC105-1wt.%Sb, SAC105-1wt.%Bi-1wt.%Sb, and SAC105-1wt.%Bi-1wt.%Sb-0.4wt.%Ti solder alloys were investigated. The following conclusion could be drawn from this study:

1. The additions of Bi, Sb, and Ti slightly reduce the T_onset_ of cooling of the solder alloy. However, the effect of adding those alloying elements on the T_peak_ of heating is negligible. When Bi, Sb, and Ti are simultaneously added into SAC105, both the undercooling and pasty range of SAC105 alloys are reduced, which is beneficial to forming reliable solder joints.
2. The wettability tests performed on Cu substrate show that adding 1wt.% of Bi or Sb improves the wettability of SAC105 alloys. The simultaneous addition of Bi and Sb shows an even stronger effect in improving the wettability. Adding Ti into SAC105-1Bi-1Sb alloy slightly reduces the wettability. Nevertheless, SAC105-1Bi-1Sb-1Ti alloy still shows better wettability than that of SAC105.
3. When 1 wt.% of Bi or Sb is added into SAC105, Bi and Sb would be dissolved into the Sn matrix without forming any Bi- or Sb- containing precipitates. The matrix is uniformly distributed with a reticulated eutectic region consisting of noddle-like Ag3Sn and dark gray Cu6Sn5. The grain size of β-Sn is refined with the addition of Bi or Sb. And Bi exhibits a stronger refinement effect than that of Sb. Further introducing 0.4 wt.% Ti into SAC105-1Bi-1Sb alloy induces the formation of needle-shaped Ti_2_Sn_3_ intermetallic phase dispersed in Sn matrix.
4. The tensile strength of SAC105 was improved from 27.98 MPa to 40.78 MPa and 39.88 MPa by adding 1 wt.% of Bi and Sb, respectively. Meanwhile, the ductility of the alloys is reduced. The loss in ductility by adding 1 wt.% of Sb is smaller than that of adding 1 wt.% of Bi. Sample SAC105-1Bi-1Sb shows further enhanced strength of 42.40 MPa. When Bi, Sb, and Ti are simultaneously added into SAC105, the tensile strength could be further improved to 50.66 MPa. Meanwhile, the ductility could be kept at an acceptable level (>20%). The nano-indentation tests demonstrate the simultaneous additions of 1 wt.% of Bi, Sb, and 0.4 wt.% Ti could obviously improve the creep resistance of SAC105 alloy. The evident improvement of the mechanical properties could be attributed to the synergistic alloying effects of Bi, Sb, and Ti, which contribute to the improvement of mechanical properties via both solid solution strengthening and precipitation-strengthening effects.
5. The current study demonstrates that the mechanical properties, especially the creep properties, could be greatly improved when Bi, Sb, and Ti are simultaneously added into the SAC105 alloy. However, it should be noted that the creep properties were determined using the nano-indentation test, which is a micro-scale test method. Although the nano-indentation test has been widely used in characterizing the creep properties of solder materials, conventional creep tests at both ambient and higher temperatures would be considered to comprehensively evaluate the creep properties of the solder alloys.

## Figures and Tables

**Table 1:** Chemical compositions of solder alloys prepared (wt.%).

Alloys	Ag	Cu	Sb	Bi	Ti	Sn
SAC105	1.0	0.5				Bal.
SAC105-1Sb	1.0	0.5	1.0			Bal.
SAC105-1Bi	1.0	0.5		1.0		Bal.
SAC105-1Sb-1Bi	1.0	0.5	1.0	1.0		Bal.
SAC105-1Sb-1Bi-0.4Ti	1.0	0.5	1.0	1.0	0.4	Bal.

**Table 2:** Melting properties of SAC solder alloys (°C).

Alloys	Cooling T_onset_ (°C)	Heating T_onset_ (°C)	Heating T_peak_ (°C)	Undercooling (°C)	Pasty Range (°C)
SAC105	214	206.6	225	7.4	18.4
SAC105-1Bi	211.2	202.8	223.9	8.4	21.1
SAC105-1Sb	212.2	211	224	1.2	13
SAC105-1Bi-1Sb	211.7	200	225.5	11.7	25.5
SAC105-1Bi-1Sb-0.4Ti	212.6	212.2	226	0.4	13.8

**Table 3:** Size of β-Sn of SAC solder alloys.

Alloys	β-Sn (μm)
SAC105	64.532 ± 21.682
SAC105-1Bi	44.963 ± 18.198
SAC105-1Sb	56.872 ± 21.78
SAC105-1Bi-1Sb	42.296 ± 21.63

**Table 4:** Mechanical properties of solder alloys with different compositions.

Component	Ultimate Tensile Strength (MPa)	Yield Strength (MPa)	Total Elongation (%)
SAC105	27.98 ± 2.68	25.85 ± 1.73	48.75 ± 5
SAC105-1Sb	39.88 ± 3.82	27.74 ± 2.64	34.52 ± 6.68
SAC105-1Bi	40.78 ± 3.88	31.71 ± 4.08	27.41 ± 4.22
SAC105-1Bi-1Sb	42.40 ± 5.08	30.55 ± 3.66	22.93 ± 4.00
SAC105-1Bi-1Sb-0.4Ti	50.66 ± 1.64	35.28 ± 1.32	21.35 ± 6.33

**Table 5:** The atomic radius of different alloying elements.

Elements	Sn	Bi	Sb	Ti
Atomic radius(pm)	140	146	141	132

## Data Availability

Not applicable.
